# Comparative Evaluation of Various Extraction Techniques for Secondary Metabolites from *Bombax ceiba* L. Flowering Plants along with In Vitro Anti-Diabetic Performance

**DOI:** 10.3390/bioengineering9100486

**Published:** 2022-09-20

**Authors:** Sara Yasien, Muhammad Muntazir Iqbal, Mohsin Javed, Maha Abdallah Alnuwaiser, Shahid Iqbal, Qaiser Mahmood, Eslam B. Elkaeed, Ayed A. Dera, Hamad Alrbyawi, Rami Adel Pashameah, Eman Alzahrani, Abd-ElAziem Farouk

**Affiliations:** 1 College of Pharmacy, University of the Punjab, Lahore 54590, Pakistan; 2 Department of Chemistry, School of Science, University of Management and Technology, Lahore 54770, Pakistan; 3 Department of Chemistry, College of Science, Princess Nourah bint Abdulrahman University, Riyadh 11671, Saudi Arabia; 4 Department of Chemistry, School of Natural Sciences (SNS), National University of Science and Technology (NUST), H-12, Islamabad 46000, Pakistan; 5 Chemistry and Chemical Engineering Guangdong Laboratory, Shantou 515031, China; 6 Department of Pharmaceutical Sciences, College of Pharmacy, AlMaarefa University, Riyadh 13713, Saudi Arabia; 7 Department of Clinical Laboratory Sciences, College of Applied Medical Sciences, King Khalid University, Abha 61421, Saudi Arabia; 8 Pharmaceutics and Pharmaceutical Technology Department, College of Pharmacy, Taibah University, Medina 42353, Saudi Arabia; 9 Department of Chemistry, Faculty of Applied Science, Umm Al-Qura University, Makkah 24230, Saudi Arabia; 10 Department of Chemistry, College of Science, Taif University, Taif 21944, Saudi Arabia; 11 Department of Biotechnology, College of Science, Taif University, Taif 21944, Saudi Arabia

**Keywords:** *Bombax ceiba* L., flower, polyphenols, flavonoids, anti-diabetic activity, yeast

## Abstract

*Bombax ceiba* L. (Family: Malvaceae) was rightly called the “silent doctor” in the past as every part of it had medicinal value. For centuries, humans have used this plant according to the traditional medicinal systems of China, Ayurveda, and tribal communities. Recently, with an emerging interest in herbals, attention has been paid to scientifically validating medicinal claims for the treatment of diabetes using secondary metabolites of *B. ceiba* L. flowers. In the present study, specific secondary metabolites from the flowers of *B. ceiba* L. were isolated in good yield using the solvent extraction methodology, and their in vitro anti-diabetic efficacy was examined. Extraction efficiency of each solvent for secondary metabolites was found in following order: water > ethanol> methanol > chloroform > petroleum ether. Quantitative analysis of secondary metabolites showed 120.33 ± 2.33 mg/gm polyphenols, 60.77 ± 1.02 mg/g flavonoids, 60.26 ± 1.20 mg/g glycosaponins, 0.167 ± 0.02 mg/g polysaccharides for water extract; 91.00 ± 1.00 mg/g polyphenols, 9.22 ± 1.02 mg/g flavonoids, 43.90 ± 0.30 mg/g glycosaponins, 0.090 ± 0.03 mg/g poly saccharides for ethanol extract; 52.00 ± 2.64 mg/g polyphenols, 35.22 ± 0.38 mg/g flavonoids, 72.26 ± 1.05 mg/g glycosaponins, 0.147 ± 0.01 mg/g polysaccharides for methanol extract; 11.33 ± 0.58 mg/g polyphenols, 23.66 ± 1.76 mg/g flavonoids, 32.8 ± 0.75 mg/g glycosaponins, 0.013 ± 0.02 mg/g polysaccharides for chloroform extract; and 3.33 ± 1.53 mg/g polyphenols, 1.89 ± 1.39 mg/g flavonoids, 21.67 ± 1.24 mg/g glycosaponins, 0.005 ± 0.01 mg/g polysaccharides for petroleum ether extract. Glucose uptake by yeast cells increased 70.38 ± 2.17% by water extract.

## 1. Introduction

Many parts of *Bombax ceiba* L., such as flowers, stem bark and leaves, have proven to be hypoglycaemic in animal models, and root powder was proven to be anti-diabetic for type 2 patients in 2008 [1,2,3,4,5]. This plant has been called the “silent doctor” because each part has been used widely by herbalists as medicine. The paste of flowers and leaves is also employed for diseases related to the skin [6,7,8,9]. Other ethnomedicinal uses are in the treatment of haematuria, cancer, snake bites, sores, boils, anaemia, premature ejaculation, menstrual disorders, hydrocoele, permanent sterilization, gonorrhoea and colitis and as a laxative, diuretic and astringent [10,11,12,13,14,15]. Ayurveda preserves the traditional remedies of different parts of *Bombax ceiba* L. For example, demulcent and tonic uses are associated with the roots, gum and bark [2]. Prickles are used to enhance skin colour. Seeds are employed as medicine for the treatment of gonorrhoea [16]. The bark is used for improving the colour of skin. People of the Purulia district use leaves of *Bombax ceiba* L. for intestinal worms [17,18,19].

Various researchers have found a variety of useful compounds in the flowers of *Bombax ceiba* L.: scopolamine, protocatechuic acid, esculetin, isomangiferin, mangiferin, isovitexin, vitexin, rutin, chlorogenic acid, methyl chlorogenate, vanillic acid, quercetin, fraxetin [20], palmitic acid, ethyl palmitate, β-sitosterol [21], (2R, 3R, 4R, 5S)-5-(6-(2,3-dimethylbutyl)-7-hydroxy-2-(4-hydroxyphenyl)-2H-chromen-5-yloxy)-6-methyl-tetrahydro-2H-pyran-2,3,4-triol [22], quercetagetin-3-O-D-glucofuranoside from gynaceum [23], pelargonidin-5-β-glucopyranoside and cyanidin 7-methyl ether -3-β- glucopyranoside from petals [24], polysachharides [25], bombalin, bombasin, bombasin 4-O-β-glucoside, anthocyanin A, anthocyanin B [26], vicinine-2 and Apigenin [27,28].

Naturally occurring secondary metabolites like polyphenols, flavonoids and saponins in plants show a significant potential against diabetes. Basically, the onset of hyperglycaemia leads to diabetes mellitus, which starts from the disturbance in the metabolism of glucose [29,30,31]. Polyphenols from plant sources have shown an effective role against diabetes in animals. It has been reported that flavonoids stimulate the release of insulin and enhance Ca^2+^ uptake from islet cells [32,33,34,35]. More than 800 plants are mentioned in books and journals as having a positive effect on diabetic suffering, and more plant species are being evaluated for anti-diabetic potential. The research is ongoing for the development of safer and better treatment of this ailment. Herbal medications are found to influence β-cells positively and regulate insulin levels [36]. The tribal history of many civilizations supports the use of *Bombax ceiba* L. powder for diabetes treatment [37].

The above discussion shows the magnitude of traditional value found in *Bombax ceiba* L. and its use as a remedy for several diseases. In the present study, the red silk cotton tree (*Bombax ceiba* L.), locally famous as the “sumbal tree”, was selected. Considering the importance of this plant, this study attempts to extract the secondary metabolites from the flowers of *Bombax ceiba* L. using a solvent extraction strategy. Several solvents are employed in this study for the extraction of secondary metabolites in cold and hot conditions. FTIR and UV-vis spectroscopic analysis of the extracts was performed to analyse the specific functional group in the metabolites. Subsequently, a quantitative determination of the secondary metabolites, including polyphenols, flavonoids, glycosaponins, and polysaccharides, was carried out, and an in vitro model of anti-diabetic potential was optimized for all of the flower extracts.

## 2. Materials and Methods

### 2.1. Collection and Identification of Plant Material

In the present study, the red silk cotton tree (*Bombax ceiba* L.), locally famous as the “sumbal tree”, was selected. The fully blossomed flowers of *Bombax ceiba* L. were collected in March from the University College of Pharmacy, University of the Punjab, Lahore, Pakistan, from different trees in the front garden. The flowers were cleaned and shade-dried for 30 days. Further flowers were dried in an Anton Paar oven at 40 °C for three days. Dried flowers were sieved, pulverized and stored in air-tight containers [38].

### 2.2. Chemicals

Methanol, chloroform, petroleum ether, hydrochloric acid, acetic acid and glucose were purchased from BDH, Poole, UK. Ethanol, hexane, acetone, and water were purchased from Fisher Scientific, Hampton, NH, USA. Quercetin and anthrone reagent were provided by Sigma Life Science, Darmstadt, Germany. Gallic acid was provided by Sinochem, Beijing, China. Aluminium nitrate was purchased from Merck A.G, Darmstadt, Germany. Yeast produced by the Rose Pair company was purchased from Moon Market, Iqbal Town, Lahore, Pakistan. Folin and ciocalteus phenol reagent were from Unichem Chemicals, Dublin, Ireland.

Extractions were performed at room and elevated temperatures, named as cold extraction and hot extraction, respectively. For hot extraction, petroleum ether, chloroform and methanol were used as a solvent for extraction in a sequence relative to their increasing polarity order at a temperature slightly lower than their boiling points. In the case of cold extraction, the first three times, ethanol was used as a solvent for extraction. Then, in the same manner, extraction was done with water for the material left after the ethanol extraction. After evaporation of all the volatiles, the yields of each extraction were calculated and are given in Table 1. The names given to the sample are according to the solvent used for the extraction. PBC, CBC, MBC, EBC, and WBC stand for the extraction of *Bombax ceiba* flower in petroleum ether, chloroform, methanol, ethanol, and water, respectively. The high yield of water and methanol shows their higher extractive values, which means these solvents have shown more potential to dissolve and extract the compounds from the defatted floral powder of *Bombax ceiba* L. than petroleum ether, chloroform and ethanol. This finding can also be interpreted as the presence of more polar compounds in the defatted floral powder of *Bombax ceiba* L. than non-polar compounds because of the solubility rule of “like dissolves like”.

### 2.3. Hot Extraction Method

Hot extraction was performed using the Soxhlet apparatus. Solvents were used in increasing order of their polarity, i.e., petroleum ether (B.P 40–60 °C) first, then chloroform (61–62 °C), and lastly methanol (64–65 °C). For this purpose, 430 g of dry floral powder was packed in a filter paper bag of appropriate capacity, and approximately 3.5 L of petroleum ether was added. The temperature of the apparatus was kept below the boiling point of the solvent. All the solvents were evaporated using a rotary evaporator. The extracts were concentrated to nearly 100 mL of thick fluid, and for further removal of leftover solvent, the liquid extracts were dried in the oven at 50 °C. The extracts were weighed repeatedly to observe whether a constant mass was achieved. When the remaining mass became constant, it showed that there is no more solvent to be evaporated; then, the semi-solid or solid masses were removed from the oven. The extracts were weighed, and the % yield was calculated regarding initial weight. The extracts were stored at room temperature in air-tight bottles and away from direct exposure to sunlight.

### 2.4. Cold Extraction Method

Twenty-five grams of dry floral powder with 150 mL of ethanol in a 1000 mL beaker was allowed to be stirred. The beaker was covered with aluminium foil to avoid loss of solvent. After 1 h, the solvent was decanted and filtered. Another 150 mL of ethanol was added to the same powder and stirred over the magnetic plate for 1 h. The solvent was again collected. Another 150 mL of ethanol was added, and the filtration procedure was repeated. The three ethanol filtrates were combined in the same place. After ethanol filtrates, the left over powder material was mixed with 150 mL distilled water, stirred for 1 h and filtered. All the solvents were evaporated using a rotary evaporator. The extracts were concentrated to nearly 100 mL of thick fluid, and for further removal of leftover solvent, the liquid extracts were dried in the oven at 70 °C. The extracts were weighed repeatedly to observe whether a constant mass was achieved. When the remaining mass became constant, it showed that there is no more solvent to be evaporated; then, the semi-solid or solid masses were removed from the oven. The extracts were weighed, and the % yield was calculated regarding initial weight. The extracts were stored at room temperature in air-tight bottles and away from direct exposure to sunlight.

### 2.5. Organoleptic Characterization

Organoleptic properties of extracts, i.e., colour, smell, consistency and physical state, were noted and observed throughout experimentation to ascertain physical stability.
**Sr. No.****Sample****Physical Characteristics****Sign of Instability**1.PowderLight brown and amorphousNo Apparent Change2.PBCYellowish green semi-solid with petroleum smellNo Apparent Change3.CBCGreenish semi-solid with characteristic smellNo Apparent Change4.MBCDark brown semi-solid with no smellNo Apparent Change5.EBCLight brown semi-solid with no smellNo Apparent Change6.WBCDark brown semi-solid with no smellSemisolid to Solid

### 2.6. The General Procedure of Determination of Total Polyphenolic Contents

Total polyphenols were found using the Singleton (1965) method, in which 1 mg/mL solution in water or methanol was prepared for Gallic acid and extracts. These were standard stock solutions of Gallic acid and standard solutions of extract, respectively. The procedure was as follows. Take 10 µL from Gallic acid solution, add 990 µL distilled water. Take 20, 40, 80, 120 µL from gallic acid stock solution separately and make up each dilution to 1000 µL with distilled water. Take 200 µL from each dilution and add to it 200 µL FC reagent and 1 mL of 15% Na_2_CO_3_ and then add methanol to make the final volume 3 mL. For extracts, take 200 µL from extract stock solution, 200 µL FC reagent and 1 mL of 15% Na_2_CO_3_ and make 3 mL volume with methanol. For the blank, 200 µL of methanol in place of the extract is used. Allow standing for 90 min in the incubator at room temperature. Absorbance is measured at 760 nm. Draw the calibration curve for the standard. The concentration of total phenolics in extracts is read through the standard curve and expressed as mg of Gallic acid per gram of extract. The procedure is repeated for each extract separately and readings are taken in triplicate.

### 2.7. The General Procedure of Determination of Total Flavonoid Contents

Quercetin was used as a standard antioxidant. A stock solution of 1 mg/mL quercetin was diluted to 10 µg/mL, 20 µg/mL, 40 µg/mL, 80 µg/mL and 120 µg/mL using analytical grade methanol as a solvent. Various extracts were prepared with methanol of 1 mg/mL strength. To 200 µg/mL of each strength of quercetin, 100 µL of 10% Al(NO_3_)_3_, 100 µL of 1 M CH_3_COOH and 4.6 mL of distilled water were added. The same reagents were added to 200 µL of extract. The samples were allowed to stand for 45 min at 25 °C. The absorbance was measured by a spectrophotometer at 415 nm. The standard curve was drawn for quercetin, and the results were calculated. The procedure was repeated for each extract separately, and readings were taken in triplicate.

### 2.8. The General Procedure of Determination of Total Glycosaponin Contents

One gram of extract was carefully weighed and dissolved in 50 mL methanol in the round bottom flask and refluxed for 30 min. The filtrate was collected, and the process was repeated to obtain a second filtrate. Both filtrates were collected and reduced to 10 mL in a rotary evaporator. This 10 mL was drop-wise added to 50 mL acetone. Saponins settled down and precipitates were collected and dried at 100 °C in the oven until a constant weight was obtained. Glycosaponins were calculated using the following formula [39,40,41].

Glycosaponins = weight of ppt./weight of sample × 100

### 2.9. The General Procedure of Determination of Total Polysaccharides

Extract (200 mg) was dissolved in 7 mL of 80% hot ethanol was vortexed for 2 min and centrifuged for 10 min at 2700 rpm. Extracts were washed repeatedly until the supernatant did not give colour with the anthrone reagent. Residue was collected and dried from the water bath. Then, 25% HCl and distilled water were mixed in equal quantities (1:1). The collected residue was extracted with 10 mL of 1:1 HCl and distilled water for twenty minutes at 0 °C. The tube was subjected to 2700 rpm for 10 min in centrifuge, and the supernatant was collected in a 100 mL volumetric flask, with the volume made up with distilled water. A hundred microlitres of diluted supernatant was taken in the test tube, and 0.9 mL of distilled water and 4 mL anthrone were added and heated in a boiling water bath for 10 min; the reaction mixture was rapidly cooled, and absorbance was measured at 630 nm against the blank. The 20, 40, 60, 80, 100, and 200 µg/mL glucose solutions were used to draw the standard curve. Glucose concentration in extract was found from the standard curve and multiplied by 0.9 to obtain the total polysaccharides. The procedure was repeated for each extract separately, and readings were taken in triplicate.

### 2.10. The Procedure of In Vitro Anti-Diabetic Activity

The method of Bhutkar and Bhise (2013) was adopted with some modifications. Commercially available *Saccharomyces cerevisiae* yeast strain was washed three times and again by centrifugation (3500 rpm, 5 min) in normal saline until the supernatant became clear. Supernatants were discarded every time, and the remaining washed yeast was used to make a 10% *v/v* suspension in ice-cold water. A 100 µg/mL concentration of all extracts was prepared and added to a 10 mM glucose solution. Reaction mixtures were incubated at 37 °C for ten minutes, and then 100 µL of yeast suspension was added in all test tubes. Tubes were vortexed to allow homogeneous mixing and incubated further for one hour at 60 °C. After incubation, the reaction mixture was centrifuged (3000 rpm, 10 min). Glucose was estimated by the anthrone method in the supernatant.

$$\%\;\mathrm{Increase}\;\mathrm{in}\;\mathrm{glucose}\;\mathrm{uptake}\;=\;[\frac{\mathrm{Absorbance}\;\mathrm{of}\;\mathrm{control}\;-\;\mathrm{Absorbance}\;\mathrm{of}\;\mathrm{test}\;}{\mathrm{Absorbance}\;\mathrm{of}\;\mathrm{control}}]\times \;100$$

### 2.11. Instrumentation

An ultraviolet spectrophotometer, UV-2550, with an operating system UV probe 2.21, Agilent Technologies, Waldbronn, Germany, was used. All extracts obtained after hot and cold extraction were subjected to ultraviolet visible spectroscopy prior to experimentation. Stock solutions of 1 mg/mL strength were prepared for all extracts in distilled water or water miscible solvents (For example Methanol and DMSO). The stock solutions were diluted to prepare working solutions of 100 µg/mL strength. Scans were taken in a UV-Vis range of 200–800 nm, and λ_max_ was recorded for each against the respective methanol blank. A Fourier transform infrared spectrometer, Perkin- Elemer, USA, was used. For sample preparation, one milligram of dry floral powder was taken in a pestle and mortar to reduce its size further. Then, 100 mg of KBr was taken in a tarred crucible until constant weight was achieved and then mixed with dry floral powder in a mortar to achieve thorough mixing. A disc was prepared by compression in a hydraulic press machine, i.e., KBr disc. The spectrum was scanned in range of 4000–400 cm^−1^.

## 3. Results and Discussion

**Figure 1:**
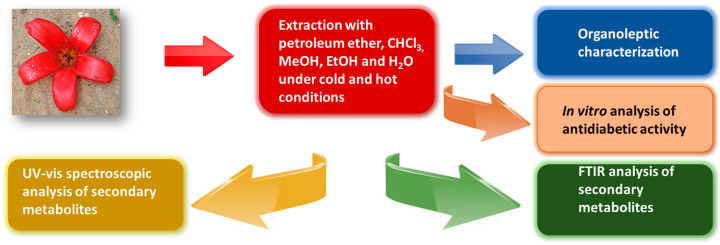
Schematic presentation of the present study.

A schematic presentation of the present study is given in Figure 1.

A schematic presentation of the current research work is helpful to understand the direction and objective of this study. It is clear from Figure 1 that after pre-treatment of *Bombax ceiba* L. flowers, multiple extraction solvents, including petroleum ether, chloroform, methanol, ethanol and water, were applied for the extraction of secondary metabolites from the subject flowers. After extraction in multiple solvents, the percentage yield of secondary metabolites in each extract was determined and is given in Table 1. Methanol and water extracts gave a greater percentage yield as compared to other solvents. Then, organoleptic characterization was performed; the detailed results are presented in Table 2.

Organoleptic characteristics were observed and recorded multiple times with naked-eye observations. Methanol and water extracts were sticky and semi-solid, while water extract turned into hard solid material as more and more water evaporated with time. Petroleum ether extract had a characteristic strong smell that was greater than the chloroform extract. Tools such as the smell and colour of extracts can be used to indicate any instability due to light, temperature, humidity and other factors. The details of organoleptic characteristics are given in Table 2.

**Figure 2:**
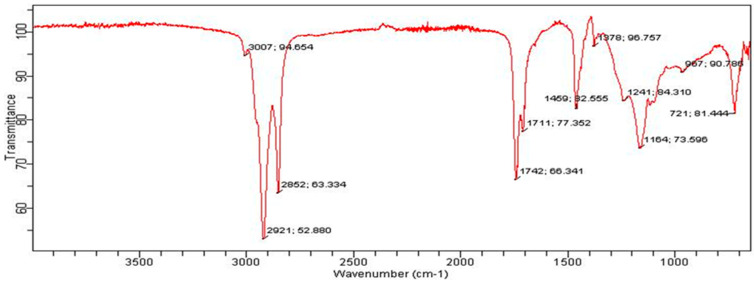
FTIR spectrum of PBC.

**Figure 3:**
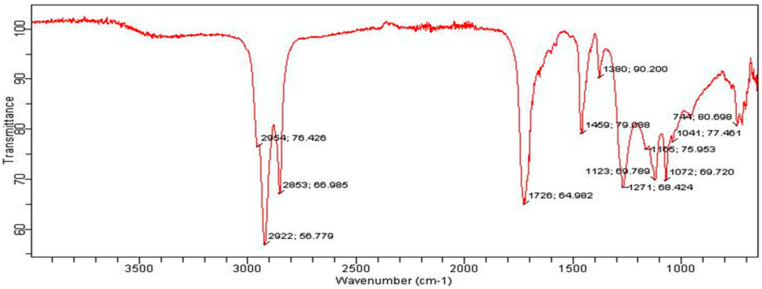
FTIR spectrum of CBC.

Next, all the extractors were analysed by FTIR spectra. The FTIR spectrum of PBC indicates the presence of aromatic groups and bending C-H vibrations at < 1000 cm^−1^, carbonyl group stretching peaks at 1742 and 1711 cm^−1^ and C-H stretching peaks at 2852 cm^−1^, 2921 cm^−1^ and 3007 cm^−1^. No peak is visible in the range of 3200–3500 cm^−1^, indicating the absence of -OH and N-H groups. The absence of -OH and N-H groups shows that hydroxyl, phenolic and amide functional groups are not present in the PBC extract. The FTIR of PBC is given in Figure 2. In the FTIR spectrum of CBC (Figure 3), the peaks that appear at 2954, 2922, and 2853 cm^−1^ indicate the presence of the –CH group. The stretching peaks at 1726 cm^−1^ identify the carbonyl group present in the extract. The peaks in the range of <1000 cm^−1^ can be ascribed to the bending vibrations of C-H and aromatic rings. A wide band in the range of 3200–3500 cm^−1^ indicates the presence of a hydroxyl or phenolic functional group in the structures of the CBC-based extract (Figure 3).

**Figure 4:**
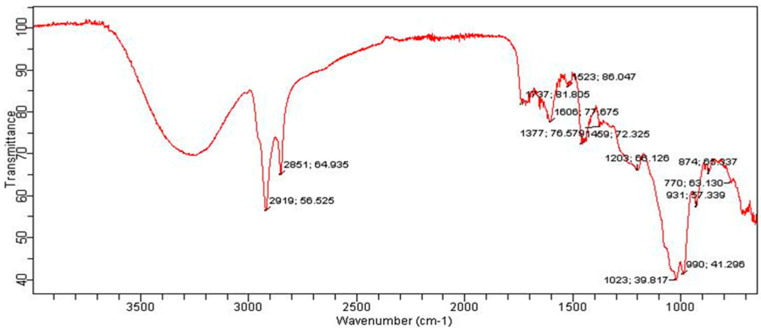
FTIR spectrum of MBC.

**Figure 5:**
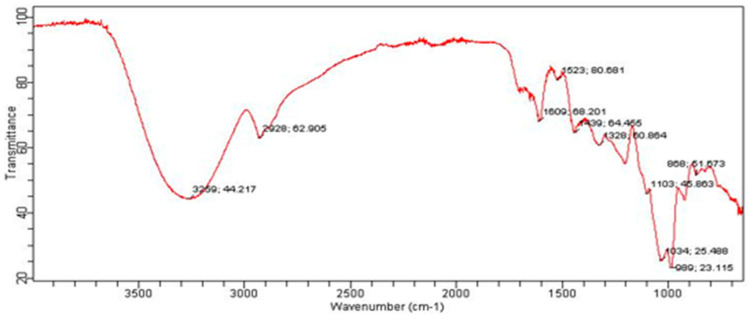
FTIR spectrum of EBC.

**Figure 6:**
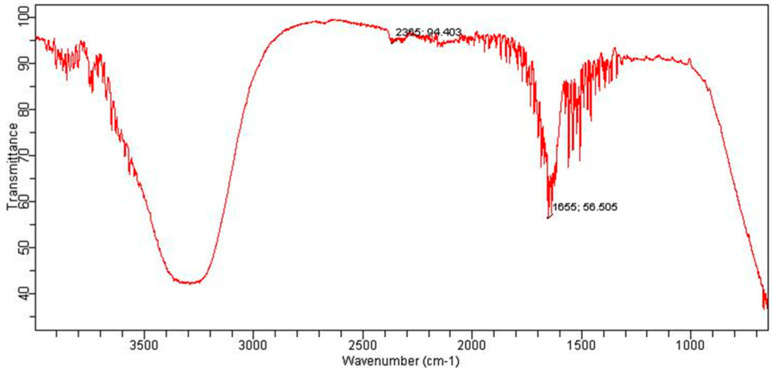
FTIR spectrum of WBC.

The FTIR spectrum of MBC is shown in Figure 4. The peaks that appeared at 2919 and 2851 correspond to the –CH group. A broad peak in the range of 3200 to 3400 is an indication of the presence of a functional group of OH or amine groups. In contrast to the FTIR spectrum of CBC, no prominent peaks for the carbonyl functional group appeared in the range of 1700, indicating the possible chance of the absence of the carbonyl group in the MBC-based extract. Similarly, the FTIR spectrum of the EBC-based extract also showed the absence of the peak for the carbonyl group (Figure 5). A wideband in the range of 3200–3500 cm^−1^ indicates the presence of hydroxyl or phenolic functional groups in the structures of the EBC-based extract (Figure 5). The spectrum of WBC showed broad peaks at around 3258 cm^−1^; this peak was again assigned to the amine or hydroxyl group (Figure 6). The peak can also be ascribed to the water, which indicates the presence of remnant water in the extract. The FTIR spectra of all the samples collectively indicated the presence of certain functional groups belonging to polyphenols, flavonoids, glycosaponins, amines, etc., in the collected extracts.

**Figure 7:**
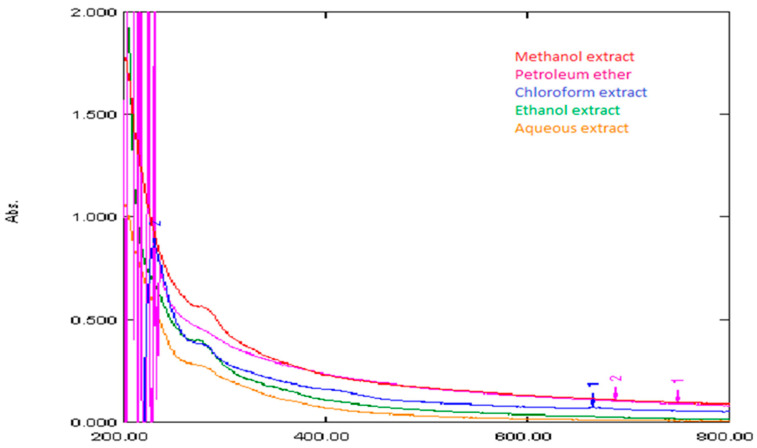
UV-vis spectra of all the extracts of the flower of *Bombax ceiba* L.

Furthermore, all the extracts were analysed by UV-Vis spectroscopy and a reference standard of each target analyte was run for quantitative measurements. The UV-visible spectra of the extracts are given together in Figure 7. The lambda maximum is found to be in the UV region. The methanol extract showed the highest peak, and the petroleum ether extract was at the lowest point of absorbance. The height of peaks follows the order of methanol > ethanol > chloroform > water > petroleum ether. The lambda maximum of the petroleum ether was at around 282 nm. All extracts showed more or less absorption in the visible region, which is also in line with the coloured nature of extracts. The deep dark brown colour of the methanol and water extract resulted in them absorbing radiation in the visible region. Water and methanol show nearly the same profile in the visible region, while chloroform and ethanol reach the same peak height in the UV region.

Subsequently, qualitative analysis was performed and determined the amounts of secondary metabolites in all the extracts. The detailed procedure for the quantitative determination of secondary metabolites is given in the experimental section. The quantitative data of secondary metabolites are given in Table 3. Quantitative analysis of secondary metabolites showed 120.33 ± 2.33 mg/gm polyphenols, 60.77 ± 1.02 mg/g flavonoids, 60.26 ± 1.20 mg/g glycosaponins, 0.167 ± 0.02 mg/g polysaccharides for water extract; 91.00 ± 1.00 mg/g polyphenols, 9.22 ± 1.02 mg/g flavonoids, 43.90 ± 0.30 mg/g glycosaponins, 0.090 ± 0.03 mg/g poly saccharides for ethanol extract; 52.00 ± 2.64 mg/g polyphenols, 35.22 ± 0.38 mg/g flavonoids, 72.26 ± 1.05 mg/g glycosaponins, 0.147 ± 0.01 mg/g polysaccharides for methanol extract; 11.33 ± 0.58 mg/g polyphenols, 23.66 ± 1.76 mg/g flavonoids, 32.8 ± 0.75 mg/g glycosaponins, 0.013 ± 0.02 mg/g polysaccharides for chloroform extract; and 3.33 ± 1.53 mg/g polyphenols, 1.89 ± 1.39 mg/g flavonoids, 21.67 ± 1.24 mg/g glycosaponins, 0.005 ± 0.01 mg/g polysaccharides for petroleum ether extract. It is observed that water extract contains the maximum concentration of polyphenols, and the lowest amount of polyphenols is found in the PBC. The overall decrease of polyphenols per gram of sample follows the order of WBC > EBC > MBC > CBC > PBC. The decreasing order of polyphenols can be ascribed to the solubility of polyphenols in the relative solvent. As water has more polar solvents, therefore, a greater amount of polyphenols is soluble in the water. The trend of polyphenol solubility can be described as “like dissolves like”. Similar behaviour was seen for the polysaccharides: the highest amount of polysaccharides are found in the water, which decreases with the polarity of the solvent used for extraction, and the lowest amount of polysaccharides is found in the petroleum ether.

In the case of flavonoids, the highest amount of flavonoids is found in the WBC extraction, followed by MCB and then CBC, and the lowest amount of flavonoids is found in the PBC. This again can be because of the solubility, as flavonoids have more polar compounds and therefore prefer to be soluble in the polar solvent. Similar results were found for the glycosaponim. The highest amount of glycosaponim is found in the case of WBC followed by MCB, and then it consistently decreases with decreasing trend of the polarity of the solvent. Contamination of herbal drugs with aerobic bacteria like streptococcus, clostridium, pseudomonas and fungi are the result of unstandardized methods of growing and storing. The chances of high microbial contamination are associated with high starch contents [42,43]. As all extracts were found to be low in contents of polysaccharides, fewer chances of microbial contamination were expected. These secondary metabolites have their particular functions for the remedies of several diseases. Polyphenols are proven as natural anti-oxidants and anti-diabetogenic compounds [44,45]. Flavonoids are polyphenolic compounds and can restore the function of the pancreas in diabetes and also reduce diabetes-induced oxidative damage [46,47].

**Figure 8:**
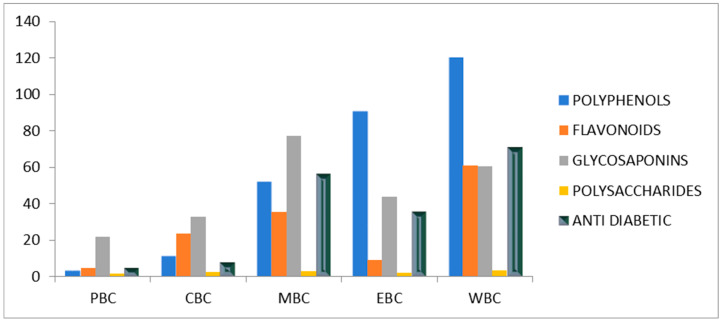
Comparison of secondary metabolites and antidiabetic activity in *Bombax ceiba* L. Note: PBC: petroleum ether extract of *Bombax ceiba* L. flower; CBC: chloroform extract of *Bombax ceiba* L. flower; MBC: methanol extract of *Bombax ceiba* L. flower; EBC: ethanol extract of *Bombax ceiba* L. flower; WBC: water extract of *Bombax ceiba* L. flower. Unit of concentration: mg/g.

To examine the in vitro antibacterial activity of all the extracts, the method of Bhutkar and Bhise (2013) was adopted with some modifications. The detail of this method is described in the experimental section. A comparison of secondary metabolites and anti-diabetic activity of the extracts is given in Figure 8. Water and methanol extract showed the maximum activity, with in vitro assay of glucose uptake by yeast cells, while petroleum ether and chloroform showed no significant activity (Table 3, Figure 8). Ethanol activity was intermediate in value. The maximum activity of water might relate to maximum extractive value and the presence of sufficient secondary metabolites.

## 4. Conclusions

In conclusion, successful solvent extraction of *Bombax ceiba* L. flowers was achieved using a simple and easy approach. The hot solvent extraction gave better performance as compared to the cold one. All the extractions were analysed by the FTIR and UV-vis spectra to obtain comparative quantitative estimation of secondary metabolites (polyphenols, flavonoids and saponins) in each extract. Quantitative analysis of secondary metabolites showed 120.33 ± 2.33 mg/gm polyphenols, 60.77 ± 1.02 mg/g flavonoids, 60.26 ± 1.20 mg/g glycosaponins, 0.167 ± 0.02 mg/g polysaccharides for water extract; 91.00 ± 1.00 mg/g polyphenols, 9.22 ± 1.02 mg/g flavonoids, 43.90 ± 0.30 mg/g glycosaponins, 0.090 ± 0.03 mg/g poly saccharides for ethanol extract; 52.00 ± 2.64 mg/g polyphenols, 35.22 ± 0.38 mg/g flavonoids, 72.26 ± 1.05 mg/g glycosaponins, 0.147 ± 0.01 mg/g polysaccharides for methanol extract; 11.33 ± 0.58 mg/g polyphenols, 23.66 ± 1.76 mg/g flavonoids, 32.8 ± 0.75 mg/g glycosaponins, 0.013 ± 0.02 mg/g polysaccharides for chloroform extract; and 3.33 ± 1.53 mg/g polyphenols, 1.89 ± 1.39 mg/g flavonoids, 21.67 ± 1.24 mg/g glycosaponins, 0.005 ± 0.01 mg/g polysaccharides for petroleum ether extract. Glucose uptake by yeast cells was increased 70.38 ± 2.17% by the water extract, while glucose uptake by yeast cells was less for other extracts. The quantitative determination of secondary metabolites showed that extraction with hot water gives the maximum extraction of secondary metabolites such as polyphenols, flavonoids, glycosaponins and polysaccharides. In vitro testing results show that water extracts of *Bombax ceiba* L. flowers increase glucose uptake by yeast cells by 70.38 ± 2.17%. Therefore, it can be used for the synthesis of anti-diabetic drugs in liquid or solid form. It would be a herbal medication for diabetic patients, without any side effects, and thus would also be economical.

## Figures and Tables

**Table 1:** Percentage yield of multi-solvent extracts of *Bombax ceiba* L. flowers.

Entry	Sample	Extract Weight (g)	Yield (%)
1	PBC	06.02	1.40
2	CBC	02.74	0.64
3	MBC	23.42	5.44
4	EBC	02.10	0.48
5	WBC	21.64	5.23

Note: PBC: petroleum ether extract of *Bombax ceiba* flower; CBC: chloroform extract of *Bombax ceiba* flower; MBC: methanol extract of *Bombax ceiba* flower; EBC: ethanol extract of *Bombax ceiba* flower; WBC: water extract of *Bombax ceiba* flower.

**Table 2:** Organoleptic properties of various extracts of flowers of *Bombax ceiba* L.

Entry	Sample	Physical Characteristics	Sign of Instability
1	Flower of *Bombax ceiba* L.	Light brown and amorphous powder	No Apparent Change
2	PBC	Yellowish green semi-solid petroleum smell	No Apparent Change
3	CBC	Greenish semi-solid with characteristic smell	No Apparent Change
4	MBC	Dark brown semi-solid with No smell	No Apparent Change
5	EBC	Light brown semi-solid with no smell	No Apparent Change
6	WBC	Dark brown semi-solid with no smell	Semisolid to Solid

Note: PBC: petroleum ether extract of *Bombax ceiba* L. flower; CBC: chloroform extract of *Bombax ceiba* L. flower; MBC: methanol extract of *Bombax ceiba* L. flower; EBC: ethanol extract of *Bombax ceiba* flower L.; WBC: water extract of *Bombax ceiba* L. flower.

**Table 3:** Quantification of secondary metabolites in different extracts of flowers of *Bombax ceiba* L.

Entry	Extract	Polyphenols (mg/g)	Flavonoids (mg/g)	Glycosaponins (mg/g)	Polysaccharides (mg/g)
1	PBC	3.33 ± 1.53	1.89 ± 1.39	21.67 ± 1.24	0.005 ± 0.01
2	CBC	11.33 ± 0.58	23.66 ± 1.76	32.8 ± 0.75	0.013 ± 0.02
3	MBC	52.00 ± 2.64	35.22 ± 0.38	72.26 ± 1.05	0.147 ± 0.01
4	EBC	91.00 ± 1.00	9.22 ± 1.02	43.90 ± 0.30	0.090 ± 0.03
5	WBC	120.33 ± 2.31	60.77 ± 1.02	60.26 ± 1.20	0.167 ± 0.02

Note: PBC: petroleum ether extract of *Bombax ceiba* L. flower; CBC: chloroform extract of *Bombax ceiba* L. flower; MBC: methanol extract of *Bombax ceiba* L. flower; EBC: ethanol extract of *Bombax ceiba* L. flower; WBC: water extract of *Bombax ceiba* L. flower.

## Data Availability

The data will be available on request.
